# Digital Maturity of Administration Entities in a State-Led Food Certification System Using the Example of Baden-Württemberg

**DOI:** 10.3390/foods14111870

**Published:** 2025-05-24

**Authors:** Sabrina Francksen, Shahin Ghaziani, Enno Bahrs

**Affiliations:** Department of Farm Management, Schloss Osthof Süd, Schwerzstraße 44, University of Hohenheim, 70599 Stuttgart, Germany; sabrina.francksen@uni-hohenheim.de (S.F.); sh.ghaziani@uni-hohenheim.de (S.G.)

**Keywords:** public administration, regional label, agri-food, digital transformation, private–public governance, European digital innovation hubs, bureaucracy

## Abstract

Digital transformation is increasingly relevant in food certification systems, improving processes, coordination, and data accessibility. In state-led certification systems, public entities hold a political mandate to promote digital transformation, yet little is known about digital maturity in these systems or how to assess it. This study assesses the digital maturity of a state-led food certification system in Baden-Württemberg, Germany, focusing on private sector stakeholders involved in its administration. Additionally, it examines potential measures that the governing public entity can take and evaluates the suitability of the methods used. A total of 25 out of 43 organisations were surveyed using the Digital Maturity Assessment (DMA) framework validated for the European Union (EU). Six dimensions were analysed: Digital Business Strategy, Digital Readiness, Human-Centric Digitalisation, Data Management, Automation and Artificial Intelligence, and Green Digitalisation. Data Management and Human-Centric Digitalisation were the most developed, highlighting strong data governance and workforce engagement. Automation and Artificial Intelligence were ranked lowest, reflecting minimal adoption but also indicating that not all dimensions might be of the same relevance for the variety of organisations. The variability in scores and organisation-specific relevance underscores the European DMA framework’s value, particularly due to its subsequent tailored consultation process and its integration into EU policy.

## 1. Introduction

Certification systems in food production are intended to support compliance with sustainability, quality, and safety standards, shaping food production through regulatory and market expectations. Although there is no comprehensive, centrally managed record or monitoring system for food labels across Europe, studies focusing on specific schemes often indicate an increase in the number of labels over time. For instance, expansions in European Union quality schemes were documented by Albuquerque et al. [1], who showed a marked rise in registered products over 25 years, and by Krystallis et al. [2], who tracked the evolution of the Designation of Origin labels from 2001 to 2014, while Nes et al. [3] found that the share of newly introduced European food products with sustainability claims and labels increased by 2.83% annually from 2005 to 2021. Also, scholarly interest in food certification has surged, with annual publications more than doubling since 2014 from about 40 to over 90 in 2022 [4]. Drivers are, among others, consumer demand for high-quality, safe food, and concerns over health, and food certification is fundamentally linked to food safety and quality, playing a key role in enhancing public health, international trade, farmers’ incomes, and overall food security [4]. Latino et al. [5] identified four key research areas: (1) certification analysis (both specific certifications like kosher and broader certification studies), (2) consumer analysis, (3) supply chain analysis, and (4) food policy and compliance. Research on food certification systems has explored governance models, stakeholder trust, and supply chain integration in contexts such as organic and geographical indication schemes [6,7]. Calls for a harmonised framework to reduce scheme complexity [5] further attest to the need for standardisation. The pilots of digital innovation in certification—such as blockchain traceability trials in fisheries [8,9] or assisted-autonomy compliance frameworks in agriculture [10]—demonstrate the technical feasibility and localised benefits of digitalisation. Nonetheless, the digital capabilities of the certifying authorities themselves remain unbenchmarked, leaving a critical practical gap.

Digitalisation within these frameworks remains relatively underexplored—as does how its level or maturity is measured. Digital transformation in the agri-food sector has been characterised by the adoption of Artificial Intelligence (AI), Internet of Things (IoT), blockchain, and big data analytics to improve efficiency, sustainability, and supply chain resilience [11,12]. The COVID-19 pandemic further accelerated such adoption, particularly in firms seeking greater traceability amid disruptions [13]. General potential barriers to certification schemes identified in the literature include time constraints, cost barriers, resource limitations, communication, and information access challenges [6,14,15,16]. However, most investigations focus on private sector pilots or broad sector analyses, leaving the digital trajectory of public, state-led food certification bodies largely unexamined.

The digital maturity assessment is widely acknowledged as a foundational step in any digital transformation process [17,18]. A variety of models exist—from socio-technical approaches in SMEs [19] to nine-dimensional frameworks for agricultural enterprises [20]. Organisations have been classified by their data management strategies into categories such as ‘paper masters’ and ‘digital champions’ [21]. Yet these frameworks have not been adapted to the strategic, governance, and automation requirements of state-led certification agencies. Further, evidence from SMEs shows that digital tools—e-forms, low-code platforms, and Building Information Modelling (BIM)—can streamline administrative workflows, yielding efficiency gains, cost reductions, and improved stakeholder satisfaction [22,23,24,25]. Despite these advances, there is no framework linking specific administrative tasks (data processing, stakeholder communication, verification) to overarching maturity dimensions in a certification context.

The governance of certification systems varies—regulatory frameworks and enforcement can be handled by private or public entities. In state-led models, public authorities hold the mandate to drive digital transformation, though coordinating a diverse array of stakeholders adds complexity. In Germany, this model plays out at the regional level, where each federal state oversees its own food quality certification system. The regional quality label of the federal state of Baden-Württemberg serves as a template for analogous schemes in other federal states like Rhineland-Palatinate, and its countrywide provision of supporting digital tools underscores its suitability as a research region for this study.

By virtue of Baden-Württemberg’s pioneering role in the conceptualization of its state quality seal and the creation of the supporting infrastructure, its certification model represents a particularly salient exemplar for our study on the impact of regional quality assurance schemes on agricultural practice. Further, in the most recent annual German Digitalisation Index, Baden-Württemberg achieved a score of 135.5—an increase of 6.5 points—sharing the top ranking with Bavaria [26]. However, no information is available on the digitalisation of the state-led certification system in Baden-Württemberg.

To effectively leverage and further develop the given potential of digitalisation, it is crucial to first assess the current digital maturity of the organisations at the core of the system. Accordingly, this study evaluates the digital maturity of private and semi-private stakeholders within a state-managed certification system using the European Digital Maturity Assessment (DMA) [27], establishing the baseline necessary for informed public governance interventions. Three key research questions are addressed:

1. What is the current level of digital maturity among private and semi-private stakeholders within the state-led certification system in Baden-Württemberg, as measured by the DMA?
2. What measures can be implemented by the public entity in the state-led certification system to close the digital maturity gaps in these organisations?
3. Is the European DMA framework an appropriate method for evaluating digital maturity and guiding policy measures within a state-led certification system?

The primary research question is answered through empirical analysis by employing the DMA. The methodology identifies gaps in the digital maturity, allows comparability across organisations, and comes with further services like cooperation with a European Digital Innovation Hub (EDIH) [27], which facilitates digital transformation. Building on these findings, the secondary questions—namely, the measures and the role of public governance as a key enabler of digital transformation in certification systems and the suitability of the chosen DMA method for assessing digitalisation in these systems—are explored in the discussion. While addressing these issues, it is essential to consider that government digital transformation projects involve many connected parts—like organisation, technology, and innovation—which makes them harder to manage [28].

## 2. Literature Review

This chapter reviews the existing literature on two central themes related to the purpose of this study. First, it provides an overview of the regulatory aspects that shape the framework and implementation of regional certification schemes. Second, it explores studies on digitalization within this context.

### 2.1. The Certification System

European publicly governed food certification frameworks, like geographical indications, traditional specialties, and optional quality marks, are organised under Regulation (EU) 2024/1143 and must or may involve public entities. In addition to these European schemes, individual regions (such as federal states) may also create their own regional labels, provided they comply with general food law requirements. These regional labels do not fall under Regulation (EU) 2024/1143. However, by notifying these programmes, they comply with the EU rules on promoting agricultural products. The ‘Guidelines for State Aid in the Agricultural and Forestry Sectors and in Rural Areas (2022/C 485/01)’, specifically paragraph 274c, address the conditions under which state aid can support agricultural quality schemes, including the requirement to follow the ‘EU best practice guidelines for voluntary certification schemes for agricultural products and foodstuffs (2010/C 341/04)’. Unlike private schemes, publicly governed certification systems operate under the principle that public administration bodies must serve the public good, as outlined in Article 10 of the Treaty of Lisbon in the EU. Accordingly, these systems must balance economic, regulatory, and social goals. Furthermore, publicly governed certification systems commonly follow a distinct structure from private schemes. Regulation (EU) 2024/1143 requires designated intermediaries, such as recognised producer organisations, to handle administrative tasks between the state, certification bodies, and producers, necessitating close coordination among stakeholders.

**Figure 1:**
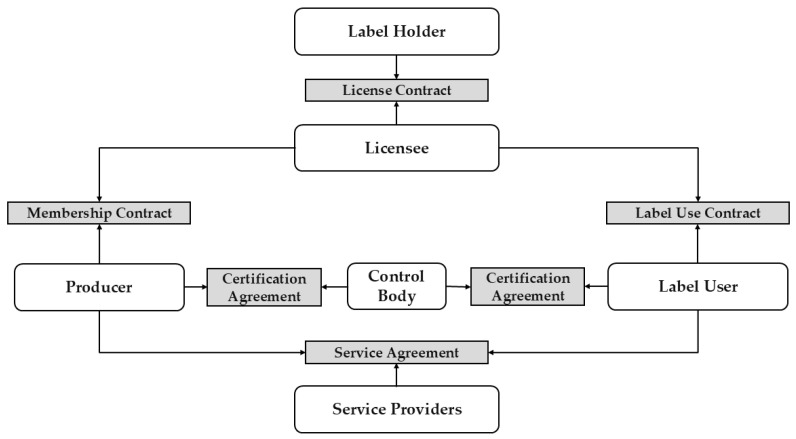
Schematic overview of the certification system of the state-led regional label of Baden-Württemberg. Shaded boxes denote the various contractual relationships between actors; unshaded boxes represent the stakeholders involved in the system. Adapted from the Ministry of Nutrition, Rural Areas and Consumer Protection of Baden-Württemberg [30].

The quality schemes examined within the scope of this study are the Quality Label Baden-Württemberg (Qualitätszeichen Baden-Württemberg, QZBW) and the Organic Label Baden-Württemberg (Biozeichen Baden-Württemberg, BIOZBW). These labels are part of a certification system established by the federal state of Baden-Württemberg, Germany, to ensure and highlight the quality and origin of agricultural products and foodstuffs. The certification schemes are governed by the Ministry of Nutrition, Rural Areas and Consumer Protection of Baden-Württemberg (Ministerium für Ernährung, Ländlichen Raum und Verbraucherschutz Baden-Württemberg, MLR) [29]. As illustrated in Figure 1, the system involves multiple stakeholders, including producers, licensees, certification bodies, and the state, represented by the MLR.

Producers (agricultural enterprises) can participate in the certification system through certification, requiring a membership contract with a licensee and a certification agreement with a certification body operating under accredited procedures and overseen by relevant authorities, adhering to Regulation (EU) 2017/625 and Regulation (EU) 2018/848. Licensees play a critical role in the system. The term ‘licensee’ typically refers to an individual or organisation granted the right to use specific properties or rights; however, in this context, it extends to include responsibilities for overseeing compliance, guiding affiliated producers, and ensuring adherence to regional quality standards within the certification system. Licensees are typically professional organisations, associations, or cooperatives within the agricultural and food industries, also called producer organisations (POs). The EU’s legal framework Regulation (EU) No 1308/2013, lastly consolidated in 2024, defines POs as entities formed and controlled by producers within specific sectors to pursue objectives such as production planning, supply concentration, and cost optimisation. These organisations may operate through various legal forms, including cooperatives, and are recognised by member states to strengthen producers’ positions in the market. Additionally, producer cooperation encompasses joint activities by producers, facilitated through recognised POs, associations of POs, cooperatives, unrecognised producer groups, and interbranch organisations, aiming to enhance collaboration and efficiency within the agricultural sector. In the certification system, licensees are responsible for implementing and overseeing the programme’s standards among their associated producers and the users of the certification mark. In contrast, the public entity and label holder, the MLR and its associated private administration body, oversee the system as a whole but do not have direct contact with the producers and users of the certification mark. The tasks of licensees include contract management, where they enter into agreements with producers and product users to ensure adherence to the programme’s quality requirements. They also monitor compliance by organising audits and inspections, manage and report data related to compliance and certifications, enforce sanctions in cases of non-compliance, and act as intermediaries between the state, producers, and other stakeholders to facilitate communication and support the continuous improvement of the certification standards. Licensees ensure the integrity of the system by managing the data flow between producers, certification bodies, and state authorities. Certification bodies are independent, neutral organisations accredited to assess compliance with the QZBW or BIOZBW standards. Their primary role is to conduct impartial audits and inspections at various points in the supply chain, including farms, processing facilities, and distribution channels. These bodies verify that producers and product users adhere to stringent product-specific requirements that exceed legal norms. Certification bodies are responsible for performing both scheduled and unannounced audits, issuing certifications that allow products to be labelled with the QZBW sign, maintaining detailed records of audits, conducting follow-up inspections in cases of non-compliance, and recommending sanctions when serious deviations from standards are found.

In summary, the certification system relies on a collaborative effort among licensees, certification bodies, and producers to maintain and enhance the quality, traceability, and credibility of agricultural products and foodstuffs in Baden-Württemberg. Licensees ensure that participants adhere to the standards, certification bodies provide independent verification, and producers implement the necessary practices to meet these rigorous requirements. The divided administrative structure leads to distributed certification responsibilities across multiple levels while maintaining oversight and compliance with the requirements set by the label holder, the MLR. The tasks within the certification system can be classified into three key areas: Data Collection and Documentation, Data Processing and Verification, and Data Flow and Communication. Data Collection and Documentation involves registering producers and product users, maintaining certification records, and gathering compliance data. Producers submit self-reported information on their processes, while administrative bodies document quality measures and monitor irregularities. Data Processing and Verification focuses on reviewing collected data, scheduling audits, and coordinating inspections. Certification bodies assess compliance, issue or renew certifications, and manage sanctions for non-conformities. Data Flow and Communication ensures smooth interactions between stakeholders. Certification bodies transmit audit results to regulatory authorities, report compliance trends to the label holder (MLR), and provide guidance to producers. Additionally, certified status updates are communicated to retailers and market participants. The literature highlights several key administrative tasks essential for ensuring food quality, safety, and compliance, including implementing robust quality-assurance models [31]. Administrative responsibilities also encompass monitoring certification bodies, supervising their organisation and management [32], and developing audit integrity systems to detect opportunistic behaviour [33].

The formal QZBW and BIOZBW framework has been emulated by other federal states. Rhineland-Palatinate, for instance, instituted its own regional certification scheme on a structure closely analogous to the QZBW. Further, the State Institute for Agriculture, Food and Rural Areas, Schwäbisch Gmünd (Landesanstalt für Landwirtschaft, Ernährung und ländlichen Raum, Schwäbisch Gmünd, LEL), conceived and maintains an integrated self-monitoring tool for agricultural enterprises [34]. This system comprises detailed checklists, document repositories, and informational leaflets, explicitly covering the QZBW and BIOZBW requirements alongside other key assurance schemes. Originally launched in Baden-Württemberg in 2002, the tool was refined from 2005 onward in collaboration with Bavaria, Rhineland-Palatinate, Hesse, and Saxony, and is also licenced by North Rhine-Westphalia’s Chamber of Agriculture. Today, state-specific versions operate in seven federal states, making it a de facto national self-control instrument.

### 2.2. Digital Maturity

While these certification systems help maintain compliance and build trust, they also increase bureaucratic complexity in administrative processes. In this light, digitalisation emerges as a promising approach to streamline these processes, enhance overall coordination, and improve data accessibility. According to McFadden et al. [35], the literature indicates that digitalisation is generally associated with improved productivity, enhanced environmental sustainability, and increased resilience in agriculture, although the extent of these benefits can differ. Anghel et al. [36] found that digital technologies can boost productivity in the medium term, provided that supportive institutional frameworks and skilled workforces are in place.

Also, regarding the specific dimensions of digitalisation, the literature finds positive effects. Automation and AI offer significant potential to streamline Data Processing and Verification in food certification. AI can enhance food safety by predicting risks, optimising supply chains, and automating compliance checks [10,37]. Machine learning and deep learning algorithms can improve food production, packaging, and quality control [38,39]. Optical Character Recognition (OCR) and classification algorithms can automate nutritional claim verification on food labels [40]. AI-powered systems can organise and analyse food safety data, providing valuable insights for decision-making [41]. Blockchain technology, combined with AI, can enhance traceability and transparency in halal food certification [42]. The integration of AI, big data, and the Internet of Things (IoT) can support early warning systems for emerging food safety risks [43]. Multi-stakeholder platforms can facilitate secure data exchange for food safety certification [44].

Digital maturity models help monitor digitalization progress in industries like conformity assessment, with technology adoption associated with higher maturity levels [45]. Decentralised approaches and blockchain technology can address trust issues in multi-stakeholder data sharing [46]. In agriculture, open data ecosystems and stakeholder interactions are crucial for digital innovation and sustainable value creation [47,48]. However, challenges remain in data-sharing practices and ecosystem maturity, particularly in sectors like agriculture [48]. Network effects and interoperability can enhance digital calibration certificate management in metrology [49]. These technologies promise to improve efficiency, accuracy, and trust in food certification processes.

While public sector digitalisation has been widely studied, e.g., regarding its status quo [50], as well as its effects [51,52], little attention has been given to the digital maturity of state-led certification systems—especially regarding their participating private and semi-private organisations that administer key certification processes.

Despite challenges, the state’s role in managing multi-stakeholder certification systems also presents significant opportunities for digital transformation. Public governance fosters trust and legitimacy, as state-led initiatives are mandated to prioritise public interest over commercial gains [53]. Further, state agencies are responsible for implementing national and EU digital strategies, like the Digital Decade Policy Program [54], at the local level. The mandate to accelerate the digitalisation of public services, in general, is also laid out in the ‘2030 Digital Compass: The European way for the Digital Decade’ [55].

According to Kurilova and Antipov [56], the implementation and development of digital technologies primarily rely on assessing the current level of digitalisation. Brodny and Tutak [57] found that evaluating the digitalisation level of companies can facilitate the creation of effective policies to support digitalisation processes. Scientific studies evaluating digitalisation typically employ methods in which the degree of digitalisation is measured across various dimensions and then aggregated into an index. Katz and Koutroumpis [58] demonstrated a positive correlation between the digitalisation index and productivity, emphasising that advances in digitalisation can lead to improved economic outcomes. One such index is digital maturity—a comprehensive concept that captures the gradual integration of digital technologies within organisations or sectors. Digital maturity refers to an organisation’s capability to leverage digital technologies for improved performance and innovation [18,59]. It encompasses multiple dimensions, including strategy, leadership, culture, and technology [59,60]. Digital maturity reflects an organisation’s capacity to effectively use technology, optimise processes, and achieve strategic goals [61]. Another definition is that digital maturity encompasses the extent to which an organisation has integrated digital technologies, processes, and mindsets into its core operations, enabling it to leverage the benefits of digitalisation effectively [62]. Thordsen et al. [63] reinforced this notion by highlighting the importance of precise measurements in digital maturity models. Over the past decade, a multitude of frameworks and models have been introduced, largely by consultancies aiming at practical implementation and each offering a unique perspective [63] to help organisations navigate their digital transformation journey [60,64]. In addition to private frameworks, academic researchers have proposed various models. Herzog et al. [19] proposed a multi-stakeholder socio-technical approach to digital transformation in SMEs, arguing that the concurrent consideration of technological artefacts, human practices, and organisational contexts can overcome the shortcomings of purely techno-centric models. The authors introduced an integrated multi-stakeholder maturity framework that synthesises human-centred job design and business-model-driven criteria, thereby enhancing both diagnostic precision and prescriptive applicability for AI-enabled work systems. Hohagen and Wilkens [20] developed a nine-dimensional digital maturity model specifically designed for agricultural enterprises, offering a diagnostic instrument to inform strategic management decisions in farm contexts. Empirically validated within a project involving 123 German farms, the instrument demonstrates advanced maturity in technological and human capital dimensions, contrasted by comparatively underdeveloped organisational dimensions. The study revealed significant heterogeneity in digital maturity across participating farms, highlighting the critical need to strengthen organisational readiness to achieve a more balanced digital transformation in agriculture [20]. One approach, originated by the EU, is the DMA framework. It was developed by the Joint Research Centre (JRC), the European Commission’s in-house scientific service, in collaboration with the Directorate-General for Communications Networks, Content and Technology (DG CNECT), the department of the European Commission responsible for EU policies on digitalisation, telecommunications, and technology innovation [27].

The DMA assesses the digital maturity of small and medium enterprises (SMEs) and public sector organisations (PSOs) using two separate questionnaires validated by JRC and tailored to the distinct organisational needs of SMEs and PSOs for effective digital transformation. This initiative is part of the broader EU Digital Europe Program (European Commission, 2021) and the EU Digital Strategy to enhance digital capabilities across Europe [55,65]. Public entities have a greater duty to adopt strategies aligned with EU objectives, as they operate within a regulatory framework shaped by European policies and are responsible for implementing national and EU-wide digitalisation goals. Unlike private entities, they must incorporate interoperability to ensure seamless integration with European digital infrastructures and cross-border digital initiatives. The questionnaires for PSOs and SMEs are nearly identical, except that the PSO version includes a section on compliance with interoperability principles. This study evaluates SMEs, and therefore, the corresponding questionnaire was employed. The DMA questionnaire designed for evaluating digital maturity within SMEs includes questions oriented around six dimensions, as described in Section 3.2.

In the present study, this method was chosen due to several key reasons. Firstly, the DMA tool provides a comprehensive evaluation of digital maturity across SMEs, addressing a broad range of businesses. This aligns well with the diverse nature of our target participants, which are small- and medium-sized companies in the sectors of retailing, processing, or cooperatives, as well as certification bodies. By utilising this framework, the companies that participate in our survey can potentially gain access to support and interventions from EDIHs, a valuable resource for driving their digital transformation. Furthermore, the fact that the DMA framework was developed and is backed by the JRC and DG CNECT lends it a high level of authority and credibility, which could be important for its potential institutionalisation within the state-led certification system.

## 3. Materials and Methods

### 3.1. Overview

**Figure 2:**
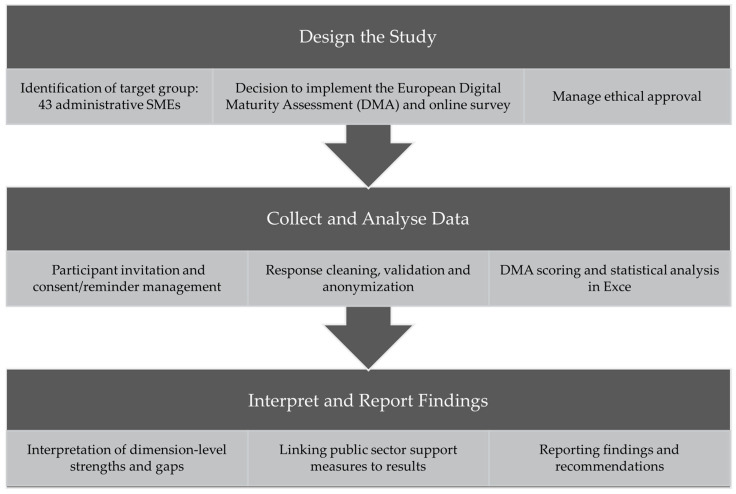
Overview of methodological steps adapted from Creswell [66].

This sub-section provides an overview of the methodological approach applied in the present study. Figure 2 illustrates the main steps undertaken to assess the digital maturity of the organisations involved in the administrative processes of food certification in Baden-Württemberg.

In the design phase, we identify our target group of 43 administrative SMEs, decide to adapt and implement the DMA as an online survey, and secure ethical approval. During data collection and analysis, we invite participants, manage consent and reminders, clean and anonymize responses, and calculate DMA scores with accompanying statistical analyses in Excel. Finally, in the interpretation and reporting phase, we interpret dimension-level strengths and gaps, link our findings to actionable public sector support measures, and present our results alongside concrete recommendations.

The following sub-sections describe each of these steps in greater detail. Section 3.2 covers the identification of the target population and the recruitment of expert participants. Section 3.3 presents the survey instrument and data collection process. Section 3.4 explains the procedures used for scoring and data analysis.

### 3.2. Survey Design

The present study evaluated the digital maturity of organisations involved in the administrative processes of food certification within Baden-Württemberg. To achieve this objective, all relevant organisations were identified, encompassing a total population of 43 organisations. These organisations include both certification bodies and licensees within the QZBW and BIOZBW certification systems. A list is publicly available on the MLR website and includes contact details [67]. The list of licensees contains 28 organisations, and the list of certification bodies contains 16 organisations. Notably, one organisation operates in a dual capacity, functioning as both a licensee and a certification body. For analytical consistency, this entity was counted in the total population as a single unit, resulting in a total of 43 unique organisations involved in the administrative processes. However, this organisation did not participate in the survey, and therefore, its dual role is not reflected in the survey results. Furthermore, there are instances where two organisations operate under the same administrative structure, sharing personnel and resources; these were also considered as a single entity. Regarding the specific certification systems, 36 organisations are involved in the QZBW system, while 17 organisations participate in the BIOZBW system. Ten organisations are involved in both systems.

All 43 identified organisations were invited to participate in an online survey. The expert participants were selected based on their strategic and administrative roles in food certification. Specifically, the aim was to recruit one representative per organisation who holds a managerial role at one of the institutions involved in the administration of QZBW or BIOZBW in Baden-Württemberg. These individuals are typically responsible for overseeing producers’ compliance with certification standards and would be involved in activities such as control and auditing or other related services. To contact them, the MLR facilitated outreach by informing the organisations about the project and inviting them to nominate an appropriate representative. Follow-up communication and formal survey invitations were coordinated through MLR, ensuring that participants were both informed and appropriately positioned to respond to the digital maturity questions.

Participation was voluntary, and the survey adhered to ethical research guidelines. The study was performed in line with the Declaration of Helsinki, and approval for the survey and methodology was granted by the University of Hohenheim’s ethics committee on 24 December 2023, under the code 2023/17_Francksen. The survey was conducted via the Enterprise Feedback Suite platform from QuestBack Unipark [68], and participants completed it independently as a self-report. To ensure compliance with data protection regulations, participants were required to review and agree to the data protection terms and provide informed consent as a mandatory requirement before proceeding with the questionnaire. To maintain participant confidentiality, the results are reported anonymously.

### 3.3. European Digital Maturity Assessment

The questionnaire used in this study was based on the DMA methodology developed by the JRC of the EU under the EDIH programme [27]. The original questionnaire, in its German version, was slightly modified and the online survey was created using the web programme Enterprise Feedback Suite Survey Version 23.1 by ist Tivian XI GmbH [68], a web-based platform for designing, distributing and analyzing surveys. Specifically, regarding the general questions about company information, irrelevant items—data points essential only for further consultation by an EDIH—were omitted. At the beginning of the questionnaire, general questions were asked, such as the organisation’s name, its function in the certification system, staff size, and information about the participating person. The remaining items were designed to assess the digital maturity of SMEs across six dimensions: Digital Business Strategy, Digital Readiness, Human-Centric Digitalisation, Data Management, Automation and Artificial Intelligence (AI), and Green Digitalisation. Table 1 shows the questions regarding each dimension of digital maturity. The complete questionnaire, including offered explanations and answer options, is provided in the DMA method document [27].

Each dimension consisted of two sub-dimensions, each represented by one question, except for Dimension 5 (Automation and AI), which had one. For example, the first sub-dimension of ‘Digital Business Strategy’ examined the enterprise’s investments in digitalisation across various business areas. This sub-dimension included questions about current and planned digital investments in specific areas such as project planning and management, administration and human resources, and cybersecurity and compliance with data protection regulations. The second sub-dimension assessed whether the enterprise had or was implementing the necessary business objectives, financial resources, Information and Technology (IT) infrastructure, and staff capabilities for digitalisation.

Similarly, the ‘Digital Readiness’ dimension evaluated the enterprise’s adoption of both mainstream and advanced digital technologies. The first sub-dimension aimed to assess the enterprise’s current adoption of a range of mainstream digital technologies and solutions that are widely used by businesses, including connectivity infrastructure, websites, web-based forms, live chats, e-commerce, e-marketing, e-government services, remote collaboration tools, and internal web portals. The question asked which of these technologies were already being utilised by the enterprise, providing insights into its current level of digital maturity and its understanding of fundamental digital tools for business operations. Moreover, the second sub-dimension of Digital Readiness examined the enterprise’s familiarity with and implementation of more advanced technologies, including simulation and digital twins, virtual reality, augmented reality, Computer-Aided Design (CAD)/Computer-Aided Manufacturing (CAM), Manufacturing Execution Systems, Internet of Things (IoT), blockchain, and additive manufacturing. Instead of merely inquiring whether the enterprise utilised these technologies, this question asked respondents to rate their engagement level with each technology on a scale from 0 to 5, where 0 indicates ‘not used’, 1 indicates ‘considering use’, 2 is ‘prototyping’, 3 is ‘testing’, 4 is ‘implementing’, and 5 is ‘operational’. This detailed approach facilitates a nuanced understanding of the enterprise’s current progress in adopting advanced digital technologies, identifying the specific level of digital development attained by the enterprise.

The ‘Human-Centric Digitalisation’ dimension focused on staff skills, engagement, and empowerment in relation to digital technologies. The first sub-dimension examined how the enterprise managed its workforce’s digitalisation skills. Participants were asked to specify actions that the enterprise had undertaken to train its staff for successful digital transformation. This included the enterprise’s strategies to address potential skill gaps, ensuring staff readiness for the challenges of a digital environment. This question aimed to assess the enterprise’s commitment to fostering a digitally competent workforce. The second sub-dimension focused on how an enterprise engages and empowers its staff when adopting new digital solutions. It explored the ways in which an organisation communicates digitalisation plans to its staff, monitors their acceptance, and mitigates potential collateral effects such as fear of change or concerns about work–life balance. The question also examined how companies involve staff in the design and development of digital solutions, provide them with autonomy and digital tools for decision-making, and adapt workflows to better suit their preferences.

The ‘Data Management’ dimension assessed the existence of a Data Management policy, the digital storage and integration of relevant data, real-time access from different devices and locations, data analysis for decision-making, and security measures in place to protect data. The first sub-dimension of Data Management explored how an enterprise manages its data by specifically asking participants to determine how their data were stored, organised, accessed, and exploited for business purposes. The aim was to understand the enterprise’s approach to Data Management, including whether they had a Data Management policy, whether data were stored digitally, and whether data were properly integrated and accessible in real-time. Additionally, the question investigated how data were analysed and reported for decision-making, whether external sources were combined with internal data for analytics, and whether data analytics were easily accessible without requiring expert assistance. The second sub-dimension assessed data security within an enterprise and aimed to gauge the level of awareness and preparedness an enterprise had regarding data security, ensuring they had robust measures to safeguard their sensitive information. It delved into the specific measures taken to protect data from cyberattacks and ensure their integrity. The questionnaire sought to understand whether the enterprise had a data security policy, if client data were protected, and if staff were trained on cybersecurity and data protection issues. It also explored how the organisation monitors cyber threats, maintains backup copies of critical data, and whether it has a business continuity plan in place to handle catastrophic failures.

The fifth dimension, ‘Automation and AI’, investigated the level of automation and intelligence integrated into business processes. Its only sub-dimension assessed the extent to which an enterprise utilised automation and AI technologies within its business processes. This question asked participants to identify and grade the specific AI and automation technologies. The grading scale ranged from 0, indicating ‘not used’, to 5, signifying ‘operational’, with intermediate values representing various stages of implementation, such as ‘consider to use’, ‘prototyping’, and ‘testing’. The question explored the enterprise’s engagement with various AI applications, including Natural Language Processing, Computer Vision, Audio Processing, Robotics, and Business Intelligence Systems. By understanding the level of adoption and implementation of these technologies, the questionnaire aimed to provide insights into the enterprise’s progress in automating and integrating AI into its operations.

Finally, the ‘Green Digitalisation’ dimension focused on the enterprise’s commitment to sustainable digital practices, including environmental considerations in digital choices and its use of digital technologies to promote environmental sustainability. The first sub-dimension utilised a multiple-choice structure, asking participants to select all applicable practices related to sustainable business models, services, products, production methods, and waste management. The question aimed to gather information about an enterprise’s current efforts in Green Digitalisation, specifically targeting practices like circular economy models, product-as-a-service offerings, usage tracking for reuse, eco-design principles, lifecycle planning, end-of-life management, sustainable materials, waste management, renewable energy sources, resource optimisation, and digital applications for promoting sustainable consumption. The second sub-dimension assessed how companies integrated environmental concerns into their digital decisions. They were rated ‘No’, ‘Partially’, or ‘Yes’ based on their dedication to eco-friendly practices in their business approaches. The questions covered whether environmental standards were part of their core model, whether they had an Environmental Management System, whether they considered the environment in technology procurement, monitored the energy usage of digital tools, and recycled old equipment. These inquiries reveal a company’s commitment to eco-conscious digital choices. The DMA method defines scoring rules for evaluating the digital maturity level based on six dimensions and eleven sub-dimensions. The next sub-section explains the methodology used to calculate the scores for each dimension based on the provided responses.

### 3.4. Data Analysis

This sub-section outlines the procedure for the analysis of data and assessing digital maturity. Data analysis and calculations were performed using Microsoft Excel Version 2504 by Microsoft Corporation [69]. The DMA framework employs a standardised scoring scale for all dimensions, ranging from 0 to 100, with higher scores indicating greater digital maturity. This scale ensures that the assessment results are comparable across different organisations, regardless of their size, sector, or location.

As explained before, the questionnaire comprised 11 questions, denoted as Q1 through Q11, designed to evaluate the various dimensions of DMA. Each dimension of DMA was represented by two questions, except for the fifth dimension, which was assessed by a single question. Accordingly, Q1 and Q2 were aligned with Dimension 1, Q3 and Q4 with Dimension 2, Q5 and Q6 with Dimension 3, Q7 and Q8 with Dimension 4, Q9 with Dimension 5, and Q10 and Q11 corresponded to Dimension 6.

These 11 questions are composed of multiple closed-ended item lists designed to assess digital maturity across the six dimensions. Each question comprises a set of items that function as indicators for a specific sub-dimension. Depending on the nature of the construct being measured, the response format varies across questions. Most questions adopt a “select all that apply” format, where respondents indicate whether a specific digitalisation-related practice or technology is present in their organisation (i.e., Q2, Q3, Q5, Q6, Q7, Q8, and Q10).

In contrast, Q1 uses a dual-checklist format, where for each item, respondents can mark either “already invested” or “plan to invest”. Selecting both options reflects a stronger engagement with that area. This format enables differentiation between past actions and future intentions regarding digital investment across ten defined business areas, such as product design, administration, logistics, and cybersecurity.

Other questions use a graded response scale, where respondents rate the implementation status of each item (i.e., Q4 and Q9 use a scale from 0 = “not used” to 5 = “operational”). Lastly, the ordinal rating format is applied in Q11, where the response options are “no”, “partially”, and “yes”, corresponding to increasing levels of implementation.

Each question is aligned with a specific dimension of digital maturity, and its items reflect various operational aspects that together represent the maturity of that dimension. For instance, in Q1 (Digital Strategy), the respondent is asked to identify whether their enterprise has already invested in or plans to invest in digitalisation across 10 core business areas, such as logistics, project management, and cybersecurity. In Q4 (Digital Readiness), a 6-point scale is used to assess the level of adoption of advanced technologies like IoT, CAD/CAM, or simulation. Q6 (Human-Centric Digitalisation) asks about practices such as flexible working arrangements, digital onboarding, or the availability of digital support teams, again using a “select all that apply” format. Similarly, Q11 (Green Digitalisation) evaluates the integration of environmental aspects into digital practices, where each item is rated as not implemented, partially implemented, or fully implemented.

These items, taken together, represent the degree to which the enterprise has adopted practices aligned with the respective sub-dimension. For more details about the items covered in each questionnaire section, including their wording and response options, see the original DMA questionnaire [6]. The questionnaire’s modular- and indicator-based structure allows the conversion of each response set into a partial score, which is then aggregated to generate a score for each of the six dimensions. These dimension scores form the basis for assessing the digital maturity of each organisation in a structured and comparable manner, as detailed below.

Focusing on the construct of the questionnaire, Q1 was composed of 10 distinct items, with respondents being provided two response alternatives for each item: ‘already invested’ or ‘plan to invest’. Scoring for Q1 was as follows: a single point was awarded for an affirmative response to either option for each item. This means that if a respondent had already invested in an item but did not intend to make further investments, they would be awarded 1 point. The same scoring applied if the respondent had not yet invested but planned to invest. If a respondent indicated both having already invested and also planning to invest in the item, they would receive 2 points for that item, signifying a stronger commitment to that aspect of digital maturity. Conversely, if neither option was selected for a specific item, the respondent would receive zero points, reflecting no engagement with that particular aspect of digital infrastructure or skills development.

Q2 featured a list of 10 items. Respondents were instructed to review these items and select all that were applicable to their enterprise. The scoring system devised for Q2 was binary, with each item selected by a respondent accruing one point to their score. This meant respondents could potentially score anywhere from 0 to 10 points on Q2, directly proportional to the number of items deemed relevant to their business operations. The score for Dimension 1 was derived by summing the total points accumulated from the responses to Q1 and Q2, according to the following equations.

$$1=\left(\sum_{Q1n=1}^{10}{ai}_{Q1n}\right)\times 3.33+\left(\sum_{Q1n=1}^{10}{pi}_{Q1n}\right)\times 3.33+\left(\sum_{Q2n1}^{10}i_{Q2n}\right)\times 3.33$$

where ‘*D*1’ is the score for Dimension 1;

‘*Q*1*n*’ denotes the *n*th item of Q1 (with ‘*Q*1*n*’ ranging from 1 to 10);

‘*ai_Q_*_1*n*_’ is the score given for ‘already invested’ as the answer to the *n*th item of Q1 (with scores being either 0 or 1);

‘*pi_Q_*_2*n*_’ is the score given for ‘plan to invest’ as the answer to the *n*th item of Q1 (with scores being either 0 or 1);

‘*Q*2*n*’ stands for the *n*th item of Q2 (with ‘*Q*2*n*’ ranging from 1 to 10);

‘*i_Q_*_2*n*_’ represents the score given for selecting the *n*th item of Q2 as relevant or irrelevant (with scores being either 0 or 1).

The score for each question was multiplied by 3.33 to scale up the overall dimension score to 100.

Regarding Dimension 2, Q3 and Q4 consisted of 10 and 7 items, respectively. In Q3, respondents were asked to select the items that applied to their enterprise. A binary scoring approach was employed for the responses; respondents were awarded one point for each item of Q3 they marked as applicable. Unlike Q3’s binary scoring, Q4 utilises a graded scoring method. Participants rated the extent of the utilisation of an item in their enterprise on a scale from 0 (not used) to 5 (operational). For compatibility with the binary scores from Q3, these responses were normalised. The scale values from 0 to 5 were converted to a uniform series of scores between 0 and 1 (i.e., 0, 0.2, 0.4, 0.6, 0.8, 1). The overall score for Dimension 2 was computed by summing the points accrued from both Q3 and Q4, as per the equation provided below.

$$D2=\left(\sum_{Q3n=1}^{10}i_{Q3n}\right)\times 5+\left[\left(\sum_{Q4n=1}^{7}i_{Q4n}\right)\times 10/7\right]\times 5$$

where *D*2 represents the score for Dimension 2;

‘*Q*3*n*’ denotes the *n*th item of Q3 (with ‘*Q*3*n*’ ranging from 1 to 10);

‘*Q*4*n*’ stands for the *n*th item of Q4 (with ‘*Q*4*n*’ ranging from 1 to 7);

‘*i_Q_*_3*n*_’ is the score given for selecting the *n*th item of Q3 as relevant or irrelevant (with scores being either 0 or 1);

‘*i_Q_*_4*n*_’ is the score based on the respondent’s grading of the utilisation of the *n*th item of Q4 (with scores being from 0 to 1 at increments of 0.2).

To ensure equal weighting for Dimension 2, scores from Q4 were multiplied by the factor 10/7, thus normalising its influence to match the 10-item Q3, thereby maintaining the balanced representation of both questionnaires in the overall score. Afterwards, the scores from both Q3 and Q4 were multiplied by a factor of 5 before being added together, allowing the combined score for Dimension 2 to scale up to a total of 100.

In assessing Dimension 3, the scores were derived from Q5, comprising 7 items, and Q6, consisting of 8 items. Participants were instructed to identify and select items that they deemed pertinent to their organisations, with each selected item yielding one point to reflect its relevance to the enterprise’s business context. The overall score for Dimension 3 was computed based on the following equation.

$$D3=\left[\left(\sum_{Q5n=1}^{7}i_{Q5n}\right)\times 10/7\right]\times 5+\left[\left(\sum_{Q6n=1}^{8}i_{Q6n}\right)\times 10/8\right]\times 5$$

where *D*3 is the score for Dimension 3;

‘*Q*5*n*’ represents the *n*th item of Q5 (with ‘*Q*5*n*’ ranging from 1 to 7);

‘*Q*6*n*’ denotes the *n*th item of Q6 (with ‘*Q*6*n*’ ranging from 1 to 8);

‘*i_Q_*_5*n*_’ and ‘*i_Q_*_6*n*_’ are the scores given for selecting the *n*th item of the respective question as relevant or irrelevant (with scores being either 0 or 1).

The raw scores from Q5 were multiplied by a factor of 10/7, while those from Q6 were adjusted with a multiplier of 10/8. This normalisation process ensured that the contributions of Q5 and Q6 to Dimension 3’s overall score were weighted equally, regardless of the differential item count in each question. Subsequently, the normalised results for Q5 and Q6 were multiplied by five and summed to yield the aggregate score for Dimension 3, which was adjusted to a maximum of 100.

In measuring Dimension 4, responses were analysed from Q7 and Q8. Q7 included eight selectable items, while Q8 comprised six. Respondents were instructed to select each item they perceived as relevant to their enterprise, with each selected item resulting in one point. The following equation shows how the score for Dimension 4 was calculated.

$$D4=\left[\left(\sum_{Q7n=1}^{8}i_{Q7n}\right)\times 10/8\right]\times 5+\left[\left(\sum_{Q8n=1}^{6}i_{Q8n}\right)\times 10/6\right]\times 5$$

where *D*4 represents the score for Dimension 4;

‘*Q*7*n*’ denotes the *n*th item of Q7 (with ‘*Q*7*n*’ ranging from 1 to 8);

‘*Q*8*n*’ stands for the *n*th item of Q6 (with ‘*Q*6*n*’ ranging from 1 to 8);

‘*i_Q_*_7*n*_’ and ‘*i_Q_*_8*n*_’ are the scores given for selecting the *n*th item of the respective question as relevant or irrelevant (with scores being either 0 or 1).

To standardise the scores from Q7 and Q8, given their different item counts, adjustments were implemented. The raw score for Q7 was scaled by a factor of 10/8, and for Q8 by a factor of 10/6. Finally, the adjusted scores from both Q7 and Q8 were multiplied by 5. The resulting values were then summed, yielding the overall score for this dimension.

Dimension 5 was assessed using a single question, Q9, which employs a graded response format. Participants rated the level of implementation for each practice on a scale from 0, indicating ‘not used’, to 5, indicating ‘fully operational’. To ensure alignment with the rest of the data, similar to Q4, this score was normalised on a scale from 0 to 1 in increments of 0.2.

$$D5=\left[\left(\sum_{Q9n=1}^{5}i_{Q9n}\right)\times 10/5\right]\times 10$$

where *D*5 is the score for Dimension 5;

‘*Q*9*n*’ denotes the *n*th item of Q9 (with ‘*Q*9*n*’ ranging from 1 to 5);

‘*i_Q_*_9*n*_’ represents the normalised score, reflecting the respondent’s perceived implementation level of the *n*th element in Q5 (with scores being from 0 to 1 at increments of 0.2).

As shown in the equation above, the Q9 score was subjected to a two-step adjustment to ensure consistency across dimensions. It was first multiplied by 10/5 for the normalisation and then by 10 to convert it to a 100-point scale, resulting in the final score for Dimension 5.

In assessing Dimension 6, two questionnaires were deployed: Q10, consisting of 10 items, and Q11, which comprised 5 items. For Q10, participants identified the items applicable to their enterprise, with each pertinent selection accruing a single point. Question 11 employed a three-level ordinal grading system whereby respondents assessed their enterprise’s integration of environmental considerations into decision-making processes and practices. The scoring ranged from 0 points for ‘no consideration’, to 1 point for ‘partial consideration’, to 2 points for ‘full consideration’.

$$D6=\left(\sum_{Q10n=1}^{10}i_{Q10n}\right)\times 5+\left(\sum_{Q11n=1}^{5}i_{Q11n}\right)\times 5$$

where *D*6 represents the score for Dimension 6;

‘*Q*10*n*’ denotes the *n*th item of Q10 (with ‘*Q*10*n*’ ranging from 1 to 10);

‘*Q*11*n*’ stands for the *n*th item of Q11 (with ‘*Q*11*n*’ ranging from 1 to 5);

‘*i_Q_*_10*n*_’ is the score given for selecting the *n*th item of Q10 as relevant or irrelevant (with scores being either 0 or 1);

‘*i_Q_*_11*n*_’ is the score based on the respondent’s grading of the consideration of the *n*th item of Q11 in their enterprise (with scores being 0, 1, and 2).

To derive the cumulative score for Dimension 6, the results from Q10 and Q11 were separately multiplied by a factor of five and then aggregated.

The overall grade for an enterprise’s digital maturity is determined by calculating the average score across all six dimensions of the assessment.

The results are presented as follows: respondent demographics, total digital maturity scores at individual and subgroup levels, and an analysis of dimensions at both aggregate and extreme-value levels. Throughout, findings are directly discussed and interpreted in the context of digital transformation within the certification system.

## 4. Results and Discussion

Table 2 provides a concise overview of the principal results, summarising respondent demographics, overall digital maturity scores, dimension-specific takeaways, and the assessed suitability of the DMA framework. The detailed results and their interpretations are discussed in depth in Section 4.1 through Section 4.4.

### 4.1. Demographics of Respondents

**Figure 3:**
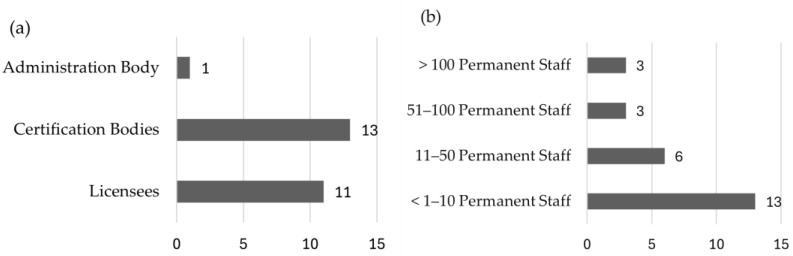
Distribution of survey respondent companies by (**a**) roles within the certification system and (**b**) permanent staff.

Of the 43 invited organisations, 25 participated in the online survey between June 2023 and July 2024, resulting in a response rate of approximately 58%. While this sample includes a substantial share of the stakeholders within the QZBW and BIOZBW certification framework, it is important to acknowledge that the total number of producer organisations, processing companies, and similar entities in the broader agri-food sector is much larger. This study focuses on the organisations involved in the administration of certification within the QZBW and BIOZBW framework, with more than half of these organisations participating in the survey. The 43 entities actively engaged in the certification system serve as the relevant reference group for assessing digital maturity. Since the primary objective is to evaluate digital maturity within the certification system and propose a framework to enhance digitalisation, the analysis centres on the stakeholders responsible for certification processes. The subgroups ‘certification bodies’ and ‘licensees’ represent distinct samples within this framework, while the overarching administration body consists of a single entity. Figure 3 illustrates the distribution of responding companies based on their roles within the certification system and their permanent staff size. Regarding roles, the largest group, licensees, comprises approximately 15 respondent companies. Certification bodies form the second-largest group, with 13 respondent companies. Since there is only one overarching administration body, only one company falls into this category. Regarding workforce size, the majority of companies (13) have fewer than 10 permanent employees. Six companies belong to the 11–50 permanent staff category, while three companies each fall into the 51–100 and over 100 permanent staff categories. This distribution highlights that most responding companies are small scale in terms of their permanent workforce.

Online survey response rates vary across research domains, with recent studies showing an average of 44.1% in education-related research [70] (Wu et al., 2022) and 42.46% in nursing research [71]. A meta-analysis of organisational research found an average response rate of 52.7% for individual-level surveys and 35.7% for organisation-level surveys [72]. Response rates have generally increased over time, from 48% in 2005 to 68% in 2020 [73]. Factors influencing response rates include participant interest, survey structure, communication methods, and privacy assurances [74]. Web-based surveys typically yield lower response rates compared to traditional methods [75]. Given these benchmarks, a 58% response rate is generally considered acceptable across different research domains, though specific context should be considered when evaluating survey reliability.

Although follow-up calls were made, the reasons for refusal were not systematically recorded and thus could not be incorporated into the analysis. Anecdotally, firms declined participation because they were ‘too small to answer these questions’, felt the survey ‘did not align with their operations’, or deemed it ‘irrelevant’, but the absence of formal data on these factors limits the ability to assess their impact on maturity scores. Future studies should collect structured non-response data and apply validity-assessment criteria or weighting adjustments to evaluate and correct for such biases. When considering the applicability beyond our study, a relevant point of discussion is regional generalizability. Data were collected exclusively from administrative entities in Baden-Württemberg, a region characterised by specific policy frameworks, funding schemes, and digital infrastructure conditions. As these institutional factors vary across federal states and countries, caution is warranted when extrapolating findings beyond this context. Comparative or longitudinal research in additional jurisdictions would be necessary to assess the broader applicability of the observed maturity profiles and support-need patterns.

### 4.2. Total Digital Maturity Scores

**Figure 4:**
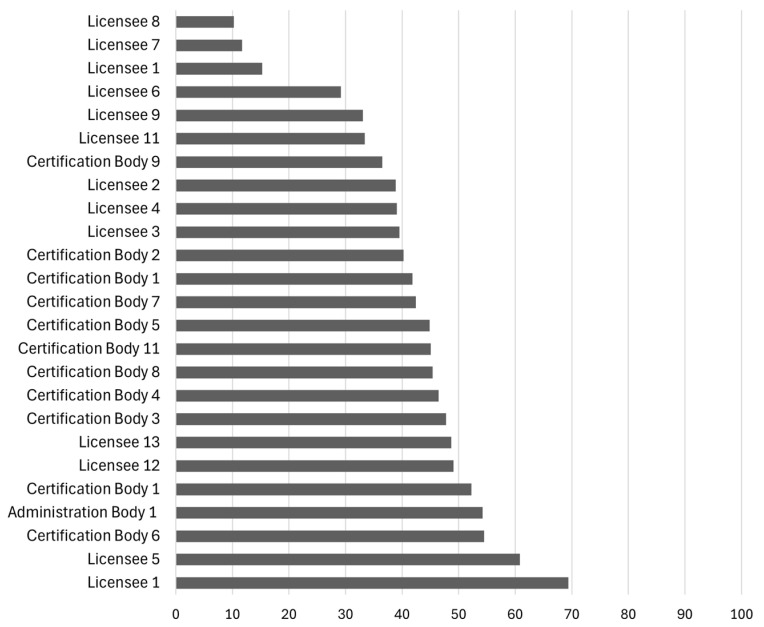
Total digital maturity scores of respondents in the exemplary certification system.

Figure 4 illustrates the individual digital maturity scores of the respondent companies, highlighting the substantial range from 10 (Licensee 8) to 69 (Licensee 1). This wide distribution suggests significant disparities in digitalisation levels, likely influenced by organisational size, resources, and digital strategies. Licensee 1, a large marketing and distribution company, stands out as the highest performer with a score of 69, reflecting a high level of digital maturity and likely a strong commitment to digital transformation. In contrast, Licensee 8, a small producers’ association, scored only 10, indicating potential challenges such as limited resources or expertise.

**Figure 5:**
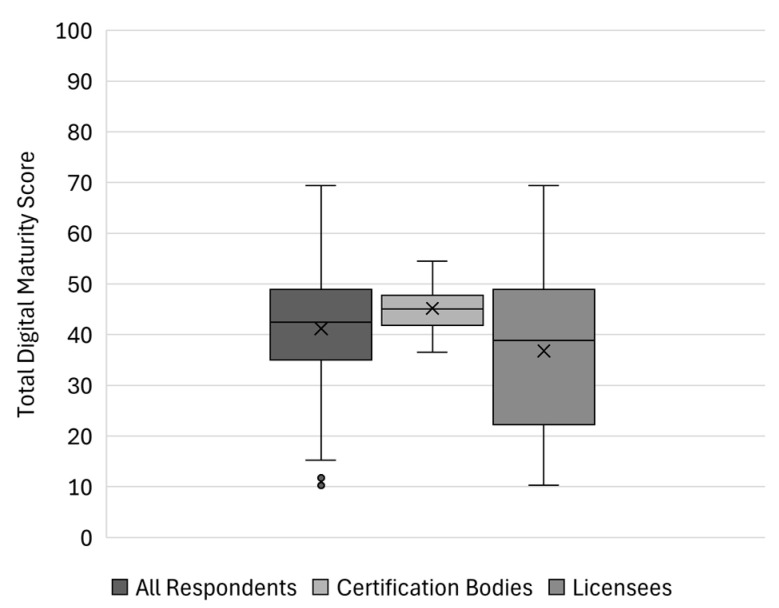
Statistics of total digital maturity scores of all respondent companies and for the subgroups certification bodies and licensees. Boxes show the interquartile range (IQR) with the median as a horizontal line; whiskers extend to 1.5 × IQR; circles denote outliers beyond 1.5 × IQR.

As shown in Figure 5, the mean digital maturity score across all respondents is 41.20, with a median of 42.43, indicating a moderate level of digital maturity within the certification system. However, a slight left skew (−0.63) suggests that some companies have lower-than-average scores, pulling the mean below the median. The standard deviation of 13.98 suggests a moderate spread of scores, with some companies performing significantly better or worse than the average. A comparison of the average total score of subgroups in the certification system, illustrated in the middle and right box plot, reveals that certification bodies exhibit higher consistency in digital maturity, with a median of 44.18 and a narrow interquartile range (40.52–46.88), suggesting more structured digital strategies. The standard deviation for certification bodies (6.91) is notably lower than for all respondents (13.98), indicating less dispersion in their digital maturity levels. Licensees, on the other hand, display the highest variability, with a median of 37.67 and a much wider interquartile range (26.84–50.88). Their standard deviation (17.17) is the highest among the subgroups, reflecting substantial differences in digital maturity within this group. The broader spread suggests that while some licensees have advanced digital capabilities, others are significantly less developed in their digitalisation efforts.

These findings highlight that while some companies within the certification system have advanced digital capabilities, others lag significantly behind. Research indicates that various factors contribute to variability in digital maturity scores within the food industry. Organisation size plays a significant role, with larger firms generally showing higher digital maturity [76,77]. Existing infrastructure and technological competence also impact Digital Readiness [78]. Specific sub-sectors within the food industry may exhibit different levels of digital maturity, influenced by factors such as customer base location and technological output [76]. Organisational culture, particularly entrepreneurial and learning-oriented cultures, is associated with higher digital maturity [79,80]. Management support and attitudes towards technology adoption are crucial factors [81]. Human factors, including employee training and participation, significantly influence technological maturity [80]. Additionally, perceived efficiency, transparency, and traceability positively affect the intention to adopt blockchain technology in the food industry [82].

The observed variability in digital maturity underscores the need for targeted digital transformation strategies to support companies with lower maturity levels, ensuring a more consistent and efficient digital infrastructure across the certification system. As previously discussed, one reason for choosing the DMA was its integration into the EDIHs consultation process, which enables tailored support based on specific organisational needs. The assumption that organisations exhibit a high degree of variability in digital maturity further strengthens the relevance of this approach. If substantial variability exists, the implementation of the DMA-EDIH process allows for structured assessments followed by customised digitalisation strategies that address individual weaknesses and priorities.

### 4.3. Dimension Scores

**Figure 6:**
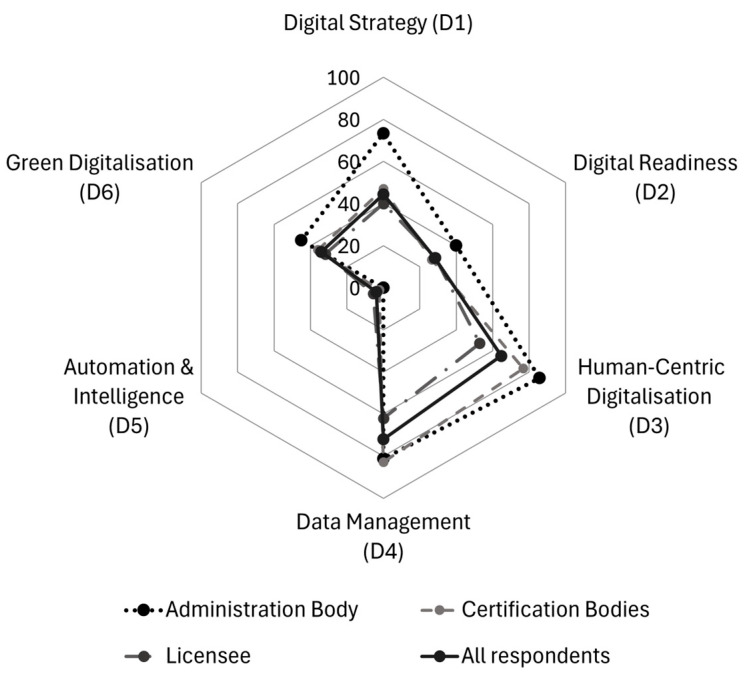
Average scores of respondent companies across six dimensions of digital transformation, classified by their roles.

Figure 6 points out the average scores of all respondent companies across six dimensions of digital transformation, classified by their roles within the certification system: administration body, certification bodies, and licensees. The digital maturity dimensions show largely consistent patterns across all respondents as well as all subgroups, with only one exception: in the administration body, Human-Centric Digitalisation (D3) scores slightly higher than Data Management (D4), whereas in certification bodies and licensees, Data Management (D4) ranks first. The overall ranking order is as follows:

(1) D4: Data Management (except in the administration body, where D3 is highest);
(2) D3: Human-Centric Digitalisation;
(3) D1: Digital Strategy;
(4) D6: Green Digitalisation;
(5) D2: Digital Readiness;
(6) D5: Automation and Intelligence.

**Figure 7:**
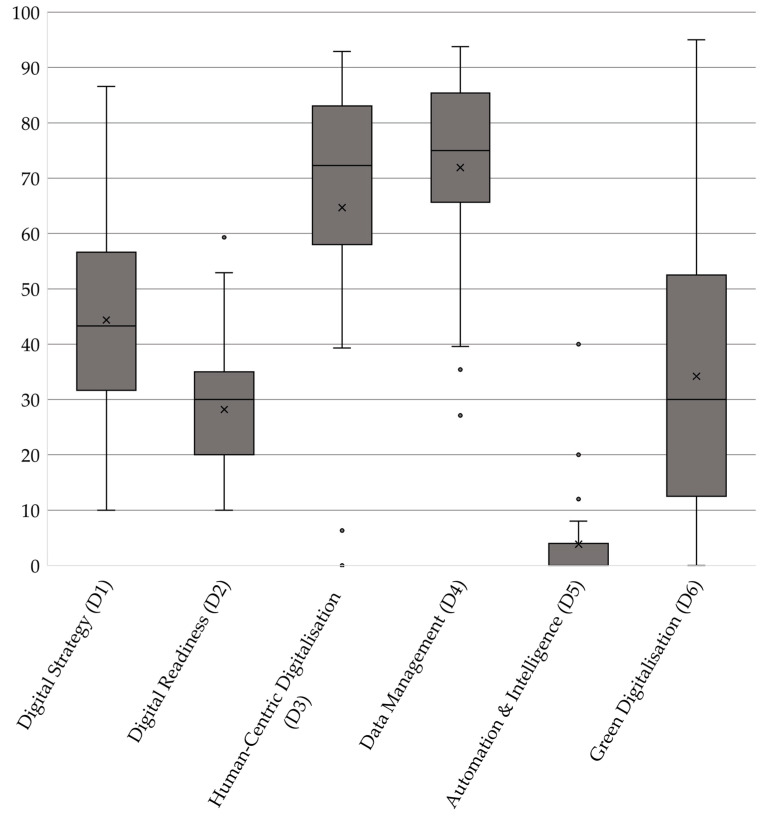
Distribution of scores of all respondent companies across six dimensions of digital transformation.

Figure 7 shows the distribution of the scores of all respondent companies across the six dimensions of digital transformation. The more detailed table, including the number of responses per question, is provided in the Supplementary Material.

Data Management (D4) emerges as the strongest dimension across all respondents, with an average score of 71.92 and a median of 75.00. The broad range of scores, from 27.08 to 93.75, suggests that while some organisations have highly structured Data Management systems, others still face challenges in data integration and utilisation. The administration body scores 81.3, demonstrating well-developed data governance and the efficient processing of compliance-related information. Certification bodies, scoring 76.79, exhibit strong data handling capabilities, likely due to their role in compliance and auditing, which necessitates well-structured data security policies, backup systems, and real-time decision support based on analytics. Licensees score 62.02, reflecting a greater variation in Data Management infrastructure. Zooming into the results, the strongest aspect in Data Management is that almost all organisations store relevant data digitally (26) and have real-time accessibility across different devices and locations (22). A significant portion also has a formal Data Management policy in place (18) and ensures the proper integration of data across different systems (19). Systematic data analysis for decision-making is moderately strong (16), showing that organisations recognise the importance of structured insights. Gaps emerge in data analytics. Only a few organisations enrich their analytics by integrating external data sources (8), and even fewer (13) make their data analytics accessible without the need for expert assistance. A notable issue is that some organisations (5) still do not collect data digitally, which hampers automation potential. Key gaps lie in the limited use of external data for analytics and the difficulty in making data insights accessible without expert knowledge. In a state-led certification system, centralised digital infrastructure and training programmes could support better data integration and usability, ensuring that all participants can leverage analytics effectively. Data security is a strong point among the surveyed enterprises. Almost all organisations have a formal data security policy (25) and ensure protection against cyberattacks for client-related data (24). Regular backups of critical business data (23) and the active monitoring of cyber threats (21) are also widely practiced, reducing vulnerabilities. Staff training on cybersecurity (20) is implemented in most cases, indicating an awareness of digital risks. Bigger gaps exist in business continuity planning. In total, 18 participants have a comprehensive plan to handle catastrophic failures, such as ransomware attacks or infrastructure damage. This suggests that while security measures are in place, resilience in crisis situations needs reinforcement. A state-led certification system could support enterprises by providing standardised frameworks and simulations for disaster recovery, ensuring that even in worst-case scenarios, operations can resume with minimal disruption.

Following closely, Human-Centric Digitalisation (D3) ranks second, with an average score of 64.68 and a median of 72.32. The wide score range (0.00 to 92.86) highlights differences in workforce engagement with digital tools across organisations. The administration body scores the highest at 85.7, emphasising strong staff engagement and empowerment, with extensive training plans, transparent digitalisation strategies, and flexible working arrangements supporting digital transformation. Certification bodies, at 76.79, display a high degree of workforce digitalisation, benefiting from structured reskilling/upskilling programmes, peer learning initiatives, and digital collaboration tools. Licensees, scoring 52.82, demonstrate considerable variation. While some companies implement digital onboarding and upskilling, others struggle with digital workforce adaptation. Overall, the strongest efforts in staff digital upskilling come from organising short training sessions, tutorials, and e-learning resources (20). Many enterprises also conduct skill assessments (17) and design structured training plans (16) to address gaps. Peer learning and hands-on experimentation (16) are used in the same proportion, highlighting a practical approach to digital adoption. External training sponsorship is offered by 15 companies, indicating some openness to external expertise. Few enterprises offer traineeships or job placements in key digital capacity areas (only two), limiting opportunities for hands-on specialisation. Additionally, subsidised training and upskilling programmes remain underutilised (7), despite being a potential resource for digital advancement. The label holder in a state-led certification system could bridge these gaps by facilitating access to funded training initiatives and encouraging structured internship programmes to build long-term digital expertise within enterprises. Regarding engaging and empowering staff when adopting new digital solutions, organisations are partly proactive. Transparent communication about digitalisation plans (21) and raising awareness of new technologies (21) are well-established practices. Staff is also involved in digital development processes (21), and flexible working arrangements (22) are widely supported, indicating a progressive approach to workforce adaptation. Job redesign and workflow adaptation to meet employee preferences (23) stand out as the most widely implemented strategies. While many companies provide digital tools for autonomous decision-making (19), fewer ensure the presence of dedicated digital support teams or services (18). Additionally, while most monitor staff acceptance (19), there is still room for improvement in mitigating concerns such as digital fatigue or privacy risks. The main gaps lie in providing structured digital support services and more targeted efforts to address staff concerns about digitalisation. In a state-led certification system, support could be offered by sharing digital support resources and developing best-practice guidelines to minimise digital transformation risks for employees.

Digital Strategy (D1) ranks third, with an overall mean of 44.36 and a median of 43.29. The score range (9.99 to 86.58) indicates disparities in strategic digital planning. The administration body, with 73.3, demonstrates a strong commitment to integrating digital strategies into product design, project management, and operational workflows. Certification bodies, scoring 46.92, reveal noticeable variations in Digital Strategy development, suggesting that while some organisations integrate digital business planning, others lack structured roadmaps. Licensees, at 39.96, exhibit the lowest levels of strategic alignment, reflecting the need for better digital investment planning and clearer long-term transformation strategies. A closer examination of overall implemented and planned investments should place a strong emphasis on whether the company’s core tasks align with the respective area. If a company does not have a primary function in a specific area, investing in digital tools for that area may be less relevant or necessary. Production-focused investment may be less relevant for service-oriented companies. The results suggest that the strongest area of digital investment is cybersecurity and General Data Protection Regulation (GDPR, Regulation (EU) 2016/679) compliance (16), reflecting a prioritisation of data protection and regulatory adherence. Operations, including manufacturing, packaging, and maintenance (15), also show investment. Collaboration with other companies in the value chain (12) and delivery logistics (12) are other notable areas where enterprises have already integrated digital solutions. Inbound logistics and warehousing (2) and purchasing and procurement (2) remain largely underdeveloped in terms of digitalisation. Similarly, marketing, sales, and customer service (10) have room for improvement, despite their role in business scalability. Encouragingly, planned investments align with these weaknesses. Inbound logistics and warehousing (9) and purchasing and procurement (9) are among the top priorities for digitalisation, indicating that enterprises recognise these areas as critical for future improvement. Marketing, sales, and customer service (9), as well as project planning and management (9), are also set for development, reflecting a growing focus on customer engagement and operational efficiency. Notably, cybersecurity and GDPR compliance (9) continue to be high-priority investments, despite already being the most advanced area, suggesting that enterprises anticipate increasing regulatory demands. Regarding readiness for digitalisation, nearly all enterprises are prepared to lead digital transformation efforts (25), and most business departments are ready to support these changes (21). Digitalisation needs are well identified and aligned with business objectives (23), and business architecture is adaptable to digital requirements (23). IT infrastructure readiness (20) and risk assessment for digitalisation impacts (20) further indicate a structured approach to transformation. However, financial preparedness is weaker—15 enterprises have secured financial resources to sustain digitalisation. Information and Communication Technology (ICT) specialist hiring or subcontracting (18) shows room for improvement, suggesting that some enterprises lack in-house expertise. Additionally, client and partner satisfaction monitoring (7) and the commercialisation of digital services (7) are minimal, indicating that external engagement and service-oriented digital models are underdeveloped. A state-led certification system could accelerate this transition by providing shared digital infrastructure or funding support for digital adoption in logistics, procurement, and customer engagement, ensuring more efficient and integrated operations across enterprises, facilitating ICT talent acquisition, and promoting digital service models to enhance competitiveness.

Green Digitalisation (D6) follows in fourth place, with an average score of 34.20 and a median of 30.00. The variability in scores (0.00 to 95.00) suggests inconsistent sustainability adoption. The administration body, scoring 45.0, integrates sustainability initiatives moderately, including circular economy approaches and energy-efficient data processing. Certification bodies, at 35.91, show mixed efforts, with some organisations implementing environmental certifications and waste reduction strategies and others demonstrating minimal commitment. Licensees, at 31.92, remain fragmented in their sustainability efforts, with some implementing paperless operations and others lacking structured environmental considerations. Enterprises primarily use digital technologies for paperless administrative processes (18), demonstrating a shift towards reducing paper waste. Sustainable service provision (15) and sustainable business models (12), such as circular economy initiatives and product-as-a-service models, are also adopted. Efforts to optimise raw material consumption (8) and reduce transport and packaging costs (8) indicate some focus on resource efficiency. Major gaps exist in sustainable energy generation (3) and waste management, emissions control (7), and sustainable production methods (6), showing that digital solutions for direct environmental impact remain underutilised. Additionally, encouraging responsible consumer behaviour through digital applications (5) is a weak point, limiting broader sustainability engagement. Regarding the implementation of environmental considerations in its digital choices and practices, only a small number have fully implemented environmental procurement criteria (6) or actively monitor and optimise energy consumption in digital technologies (3). Likewise, environmental concerns in business models (1) and the recycling/reuse of old technological equipment (2) see limited adoption. The most striking gap is the lack of Environmental Management System (EMS) implementation (20 responding ‘No’), showing that structured sustainability frameworks are largely absent. Additionally, procurement policies (15 ‘No’) and energy monitoring (15 ‘No’) remain underdeveloped, which limits proactive sustainability efforts. Enterprises show minimal commitment to environmental considerations in digital choices, particularly in structured sustainability certifications, procurement policies, and energy efficiency monitoring. A state-led certification system could support enterprises by providing incentives for clean energy adoption, promoting digital solutions for waste reduction, developing standardised digital tools for responsible consumer behaviour, promoting standardised environmental criteria in digital procurement, supporting EMS adoption, and incentivizing energy-efficient digital practices.

Digital Readiness (D2) ranks fifth, with an average score of 28.20 and a median of 30.00, showing broad discrepancies in technology adoption. The administration body, at 40.0, exhibits some technological preparedness but has gaps in advanced digital technologies such as IoT and AI-enabled solutions. Certification bodies, at 26.69, have low readiness, with basic digital infrastructures (e.g., Customer Relationship Management (CRM) and Enterprise Resource Planning (ERP)) in place but lacking integrated smart technologies. Licensees, scoring 28.57, exhibit similar trends, with limited e-commerce integration and digital connectivity challenges. The most widely adopted digital technologies include enterprise websites (26), internal web portals (25), and connectivity infrastructure (20), indicating strong foundational digital readiness. Information Management Systems (14) and remote business collaboration tools (25) are also well utilised, supporting efficient operations and workforce flexibility. The biggest gaps appear in client-facing digital tools. Few enterprises have implemented e-commerce (3), live chat or chatbots for customer interaction (6), or e-marketing (12), limiting their online sales and outreach potential. E-government services (9) are also underutilised, which could improve administrative efficiency. The Internet of Things (IoT) and Industrial IoT (two operational cases) and Manufacturing Execution Systems (two operational cases) are the only technologies in active use, though at minimal levels. One enterprise each is considering using simulation and digital twins (1) and Manufacturing Execution Systems (1), indicating some movement towards adoption. Virtual reality (22 not used), blockchain (20 not used), and additive manufacturing (20 not used) remain almost entirely absent. While one enterprise considers using simulation and blockchain, the overall awareness and experimentation with these technologies is low. A state-led certification system could encourage digital sales channels, facilitate customer interaction tools, and promote the adoption of e-government platforms to improve administrative efficiency, encouraging experimentation and structured adoption by funding pilot projects and offering best-practice frameworks to accelerate digital transformation in these areas.

Automation and Intelligence (D5) ranks the lowest, with an average score of 3.84 and a median of 0.00. This is because 19 out of 25 organisations report no use of Automation and Intelligence (median = 0), yet 7 organisations with scores from 4 to 40 raised the average to 3.84. This indicates a heavily skewed distribution, where a small number of advanced adopters contrast sharply with the majority who have little to no adoption in this area. The administration body scores 0.00, indicating that automation is not prioritised. Certification bodies, at 2.18, show a minimal use of Business Intelligence and robotic automation, likely due to the human-centred nature of certification processes. Licensees, scoring 5.54, show a few organisations experimenting with AI-driven optimisation, but most do not prioritise automation. Most organisations have not yet implemented Natural Language Processing (23), Computer Vision (23), Speech Recognition (25), Robotics (25), or Business Intelligence Systems (22). A few enterprises are testing Business Intelligence (1) and Natural Language Processing (1), while only one has moved Computer Vision into operational use. Some enterprises consider adopting AI-driven solutions, particularly in Natural Language Processing (2), Computer Vision (2), and Business Intelligence (2). However, none are actively implementing these technologies on a scale. AI and automation technologies are still in their infancy, with minimal adoption beyond isolated testing. A state-led certification system could promote AI integration by providing access to funding, training, and implementation guidelines, helping enterprises transition from consideration and testing to full operational deployment.

Assessing potential public sector measures to foster digital maturity requires evaluating their costs and benefits. Digital transformation in the agri-food sector offers significant economic benefits, including increased productivity, reduced costs, and expanded market access [83]. It can enhance decision-making, optimise resource use, and foster sustainable practices [84]. However, the process requires targeted policies, investments in digital infrastructure, and education to ensure equitable access [83]. Digital technologies can reshape market relationships within the agriculture value chain [85] and reduce trade and transaction costs [86]. Implementation barriers include resistance to change, a lack of digital skills, inadequate organisational structures, and financial constraints [87]. Despite these challenges, digital transformation presents unique opportunities for businesses to remain competitive and relevant in the evolving agri-food sector [88,89].

**Figure 8:**
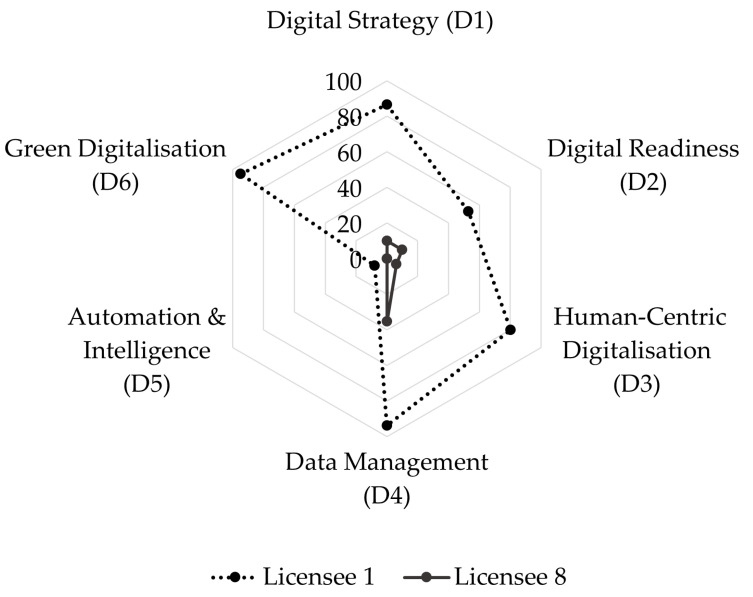
Scores of the six dimensions of the European Digital Maturity Assessment for the highest and lowest overall score.

The high variability among all dimensions and participants can also be seen when comparing the highest- and lowest-ranking participants. The radar chart in Figure 8 shows the contrast between Licensee 1 (highest overall score: 69), a large marketing and distribution company, and Licensee 8 (lowest overall score: 10), a small producers’ organisation. Licensee 1 excels in Digital Strategy, Digital Readiness, and Green Digitalisation and has moderate automation and workforce digitalisation. In contrast, Licensee 8 scores near zero in each dimension. This finding reinforces the statement that a state-led certification system should therefore emphasise the assessment of the digital maturity and the specific gaps for each enterprise.

### 4.4. Suitability of the Method

The DMA framework presents a promising approach for evaluating the digital transformation of stakeholders within a state-led certification system. However, while the DMA offers significant advantages, there are also areas that may require further refinement to ensure its full applicability for state-led certification systems. By undergoing the process of surveying participants and analysing the results in terms of their implications for necessary interventions, we observed several strengths and weaknesses in the DMA approach.

Its multidimensional structure makes it well suited for capturing the complexities of digital transformation across organisations with diverse operational contexts and varying levels of digital capability. Addressing multiple dimensions of digital maturity provides a holistic view of the current state of digital practices and challenges faced by stakeholders in certification systems. This multidimensionality ensures that critical aspects—such as technological readiness, workforce capabilities, and user-centric approaches—are all considered. The components of this assessment framework are also present, to varying degrees, in other digital maturity evaluations. This overlap suggests a common understanding of key digital readiness factors across different assessment models. However, the main weakness of the approach also lies in this broad scope. The sub-dimensions, questions, and answer possibilities may not fully align with the specific needs and functions of certification stakeholders, at least for now. E.g., investing in augmented reality might not be relevant to the stakeholders involved and their tasks in the certification systems. In some questions, the possibility is given to check ‘not applicable’, whilst others do not have this checkbox. These items may hold future importance, but being confronted with too many that seem overly futuristic could, firstly, reduce participation motivation. To reduce this risk, participants should be informed that this is a standardised survey and that some items might not be applicable to them. Second, items that are not applicable and therefore not selected still factor into the calculation as weaknesses, which could lead to an overemphasis on aspects that are not central to the stakeholders’ certification-related activities. Consequently, the results should be interpreted with a strong focus on the core aspects to avoid misallocating improvement efforts.

The insights generated by the DMA are particularly valuable for informing public governance measures aimed at fostering digital transformation. By identifying specific gaps in digital maturity, policymakers can design targeted interventions to address these deficiencies. For example, if the assessment reveals inadequate technology infrastructure among certain stakeholders, public entities could prioritise investments in connectivity or provide subsidies for acquiring essential digital tools. Similarly, low scores in workforce digital skills could prompt the development of tailored training programmes to enhance capacity building.

A major strength of the DMA is its integration with EU policies, which enhances its alignment with regional strategies, ensures long-term sustainability, and secures funding for both its direct implementation and the subsequent consultation process. This consultation cooperation with an EDIH enables stakeholders to benchmark their digital capabilities against peers and receive tailored advice on addressing company-specific challenges and weaknesses. The DMA-EDIH process can further help address a common challenge: sustaining digital assets developed in research projects beyond the funding period. Often, these assets reflect the diverse needs of multiple stakeholders, which can result in solutions that lack an addressable market of paying customers. As Aartsen et al. [90] note, public–private partnership projects frequently struggle to develop business models that ensure the long-term viability of these digital assets. In a state-led certification system, there are various possibilities on how to seize this potential. The overarching administration body could request a consultation for the system itself. Alternatively, stakeholder companies could apply for individual assessments. There is also the possibility of the label holder encouraging stakeholder participation, either by promoting the process, offering incentives for participation, or making it mandatory.

If the DMA proves to be compatible with other EU policies in the domains of data and digitalisation, it is likely to generate additional benefits for related regulations and initiatives relevant to certification systems. The Common European Agricultural Data Space (CEADS) [91] is highlighted as a central initiative that establishes a unified framework for secure and trusted agricultural data sharing, thereby promoting collaboration between public and private stakeholders. A cooperative data space—or data room—is presented as a secure network that facilitates the sharing of sensitive information and enhances process automation, data quality, and overall transparency [92]. Analogously to digital maturity, there is a need to evaluate how well organisations meet the prerequisites for participating in a CEAD. Since the DMA is meant to adapt to changes and develop in the future, there is a possibility to integrate the cooperative data perspective. An analysis identifying areas of convergence and divergence between DMA and prerequisites for a CEADS could enhance the DMA framework to assess ‘Readiness for EDS’ without increasing complexity.

## 5. Conclusions and Outlook

This study aimed to assess the digital maturity of organisations within an exemplary state-led certification ecosystem and to understand the strengths and weaknesses of organisations that are engaged in the administration process. Based on the results, measures were discussed for how the governing public entity could support these organisations in leveraging digitalization to streamline processes and improve efficiency. Furthermore, the suitability of the implemented Digital Maturity Assessment framework was evaluated.

The results indicate that organisations involved in certification have moderate-to-high digital maturity in data governance and workforce engagement but lag behind in automation, strategic alignment, and sustainability integration. The administration body demonstrates the highest maturity, with structured digital strategies and workforce training programmes. Certification bodies perform well in data-driven compliance management but show inconsistencies in digital readiness and sustainability practices. Licensees, on the other hand, exhibit the most variability, with many struggling to align digital investments with long-term transformation goals. Overall, the primary strengths lie in structured data governance (D4), effective workforce engagement (D3), and regulatory compliance through digital tools. Organisations in this sector generally prioritise secure Data Management, analytics, and digital training programmes. However, the main weaknesses include limited AI-driven automation (D5), inconsistent digital strategies (D1), and fragmented sustainability initiatives (D6). The low levels of automation adoption suggest that administration processes in the certification system remain heavily reliant on human expertise.

Integrating the organisations’ core activities into the interpretation of results highlighted that not all aspects of the DMA are equally relevant for all participants. A standardised evaluation with the DMA may highlight digital gaps, but these gaps must be adapted to operational realities, ensuring that efforts focus on the most relevant aspects rather than applying a uniform approach across all types of organisations. The high variability among participants also suggests the need for a targeted consultation process that accounts for differing organisational structures.

The implementation and discussion of the DMA process demonstrate that it aligns well with the state-led nature of the certification system and presents unique opportunities to enhance digital maturity. As a publicly governed certification system, the state can actively promote structured digital adoption by integrating the DMA-EDIH process, connecting the private organisations, and participating in administrative tasks, with technology providers, research institutions, and digital consultants utilized to strengthen weaker dimensions. By embedding digital maturity as a key criterion in public certification policies, the state could help bridge the gap between advanced and lagging organisations, ensuring a more uniform and integrated digital transformation process across the sector. The DMA survey of all stakeholders can pinpoint the most common weaknesses, enabling the public entity to centrally offer targeted solutions that benefit many organisations. For example, the public entity could establish a centralised digital training and consultancy programme focused on automation and AI, helping organisations develop these areas. When conducted regularly, the DMA can further serve as a monitoring tool that not only continuously identifies the certification system’s strengths and weaknesses but can also evaluate the effectiveness of implemented measures.

Future research should further investigate how Digital Maturity Assessments can be institutionalised in state-led certification systems and monitor how this approach helps to overcome inconsistencies in digital maturity dimensions, particularly in smaller certification bodies and licensees. More comparative studies across different sectors could provide valuable insights into best practices for adoption while keeping costs low. Additionally, further research should focus on how innovative digitalisation approaches like cooperative data use and blockchain can benefit from and/or enhance digital maturity in certification processes.

## Figures and Tables

**Table 1:** Overview of dimensions and online survey questions for the Digital Maturity Assessment.

Dimension	Question
Digital Business Strategy	In which business areas has your enterprise already invested in digitalisation and in which does it plan to invest in the future?
	In which ways is your enterprise prepared for further digitalisation?
Digital Readiness	Which digital technologies and solutions are already used by your enterprise?
	Which advanced digital technologies are already used by your enterprise?
Human-Centric Digitalisation	What does your enterprise do to reskill and upskill its staff for digitalisation?
	When adopting new digital solutions, how does your enterprise engage and empower its staff?
Data Management	How is your enterprise data managed (stored, organised, accessed, and exploited)?
	Is your enterprise’s data sufficiently secured?
Automation and AI	Which technologies and business applications is your enterprise already using?
Green Digitalisation	How does your enterprise make use of digital technologies to contribute to environmental sustainability?
	Is your enterprise taking into account environmental impacts in its digital choices and practices?

Source: Adapted from Kalpaka et al. [27].

**Table 2:** Key takeaways from survey demographics, digital maturity scores, and method suitability.

Section	Key Takeaway
Demographics	In total, 58% of participation from 43 invited certification stakeholders highlights engagement but risk of bias, with the majority of respondents being small-scale licensees.
Overall Maturity	Moderate maturity with wide gaps between most and least digitalised organisations.
D4: Data Management	Data Management is the strongest dimension with robust digital storage policies, but gaps exist in external data analytics, self-service insights, and continuity planning.
D3: Human-Centric Digitalisation	Human-Centric Digitalisation ranks second with the strong training and workforce involvement. Internships and dedicated support services remain rare despite change management.
D1: Digital Strategy	Cybersecurity is a focus, but strategic planning in logistics, procurement, and marketing lags.
D6: Green Digitalisation	Paperless admin is common, but formal sustainability frameworks are mostly missing.
D2: Digital Readiness	Digital Readiness is low with basic websites and portals; advanced tools (IoT, e-commerce, e-gov) are scarce.
D5: Automation and AI	Almost no automation or AI in daily use—just a handful of small tests.
Suitability of the DMA Method	Top and bottom performers show huge contrasts, so “one-size-fits-all” will not work.
	The framework covers all angles but includes some overly futuristic or irrelevant items.

## Data Availability

The data presented in this study are available on request from the corresponding author. The data are not publicly available due to privacy restrictions.

## Supplementary Materials

The following supporting information can be downloaded at: https://www.mdpi.com/article/10.3390/foods14111870/s1.

## Abbreviations

The following abbreviations are used in this manuscript:

AI	Artificial Intelligence
AR	Augmented Reality
BIM	Building Information Modelling
BIOZBW	Organic Label Baden-Württemberg
CAD	Computer-Aided Design
CAM	Computer-Aided Manufacturing
CEADS	Common European Agricultural Data Space
DG CNECT	Directorate General for Communications Networks, Content and Technology
CRM	Customer Relationship Management
DMA	Digital Maturity Assessment
EDIH	European Digital Innovation Hub
EFS	Enterprise Feedback Suite
ERP	Enterprise Resource Planning
EU	European Union
GDPR	General Data Protection Regulation
ICT	Information and Communication Technology
IQR	Interquartile Range
JRC	European Joint Research Centre
MLR	Ministry for Rural Affairs and Consumer Protection Baden-Württemberg
NLP	Natural Language Processing
PO	Producer Organisation
PSO	Public Sector Organisation
QZBW	Quality Label Baden-Württemberg
SME	Small and Medium Enterprises
VAT	Value Added Tax
VR	Virtual Reality
